# Reciprocally Coupled Residues Crucial for Protein Kinase Pak2 Activity Calculated by Statistical Coupling Analysis

**DOI:** 10.1371/journal.pone.0009455

**Published:** 2010-03-01

**Authors:** Yuan-Hao Hsu, Jolinda A. Traugh

**Affiliations:** Department of Biochemistry, University of California Riverside, Riverside, California, United States of America; Griffith University, Australia

## Abstract

Regulation of Pak2 activity involves at least two mechanisms: (i) phosphorylation of the conserved Thr^402^ in the activation loop and (ii) interaction of the autoinhibitory domain (AID) with the catalytic domain. We collected 482 human protein kinase sequences from the kinome database and globally mapped the evolutionary interactions of the residues in the catalytic domain with Thr^402^ by sequence-based statistical coupling analysis (SCA). Perturbation of Thr^402^ (34.6%) suggests a communication pathway between Thr^402^ in the activation loop, and Phe^387^ (ΔΔE_387F,402T_ = 2.80) in the magnesium positioning loop, Trp^427^ (ΔΔE_427W,402T_ = 3.12) in the F-helix, and Val^404^ (ΔΔE_404V,402T_ = 4.43) and Gly^405^ (ΔΔE_405G,402T_ = 2.95) in the peptide positioning loop. When compared to the cAMP-dependent protein kinase (PKA) and Src, the perturbation pattern of threonine phosphorylation in the activation loop of Pak2 is similar to that of PKA, and different from the tyrosine phosphorylation pattern of Src. Reciprocal coupling analysis by SCA showed the residues perturbed by Thr^402^ and the reciprocal coupling pairs formed a network centered at Trp^427^ in the F-helix. Nine pairs of reciprocal coupling residues crucial for enzymatic activity and structural stabilization were identified. Pak2, PKA and Src share four pairs. Reciprocal coupling residues exposed to the solvent line up as an activation groove. This is the inhibitor (PKI) binding region in PKA and the activation groove for Pak2. This indicates these evolutionary conserved residues are crucial for the catalytic activity of PKA and Pak2.

## Introduction

Statistical coupling analysis (SCA) regards evolution as a natural mutagenesis process and utilizes the known protein sequences to economically examine comprehensive correlations between amino acid residues. This analysis retrieves two co-evolved residues in a protein for either structural or functional reasons. The statistical coupling through sequence-based analysis was introduced as a reporter of thermodynamic coupling in proteins with PDZ domains [1]. Other research applied the matrix clustering analysis to systematically analyze large amounts of SCA data to identify the key residues for the structures of the G protein-coupled receptors [2], G proteins [3], and RXR heterodimers [4]. Cross-correlation analysis of sequence-based SCA and structure-based molecular dynamics predicted energetic coupling residues essential for HhaI (*Haemophilus haemolyticus* I) methyltransferase catalysis [5].

The SCA results are presented in a two-dimensional array (matrix), representing the correlation values between any of the two residues. Suel *et al.* [2] used matrix clustering analysis to find the correlation patterns from the two-dimensional SCA array. The correlation pattern showed the residues coupled reciprocally to structural determinants of the G protein-coupled receptors. We incorporated this concept and retrieved all reciprocal coupling residues of Pak2. The distilled results of the statistical coupling analysis eliminated all perturbations from a single direction. These reciprocally coupled pairs of residues are the structurally or functionally coupled residues occurring through evolution.

Pak2 belongs to the serine/threonine protein kinase family containing the conventional Pak 1–3 and Pak 4–6 [6]. Pak2 is primarily inactive during growth, and is transiently activated by Cdc42 in response to moderate stress, and constitutively activated during apoptosis by caspase cleavage [7], [8]. Pak2 has a basal autophosphorylation activity wherein five serines in the regulatory domain of Pak2 can be autophosphorylated [9]. Activation of the protein kinase requires autophosphorylation at seven serine sites in the regulatory domain and one conserved threonine in the catalytic domain [8], [10]. Cleavage by caspase 3 or binding of Cdc42(GTP) facilitates autophosphorylation at Ser^141^, Ser^165^ and Thr^402^ [11], [12]. Thr^402^ and Ser^141^ are critical for Pak2 regulation and activation [11], [12].

Inactive Pak1 exists in solution as a homodimer, with the autoinhibitory domain (AID) of each monomer interacting with its partner to inhibit activity [13], [14]. As Pak1 and Pak2 are homologous, it is generally assumed that Pak2 exists as a *trans*-autoinhibited homodimer. The bilobal structure of the catalytic domain is the conserved signature in protein kinases. Movement of the two lobes to an open or closed conformation regulates the phosphorylation efficiency in the cAMP-dependent protein kinase (PKA) and the insulin receptor [15], [16]. Pak2 catalytic activity is inhibited by binding of two α-helices in the AID (residues 111–131) to the G-helix, and the kinase inhibitory segment (KI, residues 136–149) to the active site cleft [17]–[19]. Autophosphorylation of Thr^402^ is through intermolecular phosphorylation, as opposed to the intra-molecular phosphorylation of the other 7 serine sites [20].

Post-analysis of the SCA data requires a good structural model. Although there is no Pak2 structure published, X-ray crystal structures of Pak1 are good working models for Pak2. Pak2 has 93% homology in the catalytic domain. Both the inactive (1F3M.PDB) and active conformations (1YHV.PDB) of Pak1 were utilized for analysis of Pak2 [14], [21]. The inactive conformation (1F3M.PDB) illustrates a catalytic domain with a disordered activation loop, hydrogen bonding between the Lys138 (Lys141 for Pak1) to the catalytic residue Asp368 (Asp389 for Pak1), and hydrophobic interactions between the AID and the catalytic domain [14]. The active conformation of Pak1, containing only the catalytic domain, shows a conformational change in the activation loop as compared to the inactive conformation [21].

Human protein kinases from 8 hierarchical groups with the conserved bilobal structure in their kinase domain are the perfect candidates for SCA [22]. We applied the SCA to the sequence of Pak2 (STE hierarchical group) to examine the evolutionary structural and functional determinants in the catalytic domain. Because Pak2 autophosphorylation on Thr^402^ in the activation loop is necessary for Pak2 activity, analysis of the correlative sites in the catalytic domain will define residues that co-evolved with Thr^402^. Two other protein kinases, PKA and Src (in the AGC and TK protein kinase hierarchical groups, respectively) were examined and compared to Pak2. PKA is a tetramer of two regulatory and catalytic subunits. The catalytic subunit has a similar structure to the catalytic domain of Pak2 and was used to analyze the solvent accessibility of Pak2 by H/D exchange [23]. PKA has the conserved threonine phosphorylation site in the activation loop, and the crystal structures of PKA provide good working models to study Pak2 [24], [25]. The protein tyrosine kinase Src is a monomer or a dimer, and has many published crystal structures [26]–[29]. Tyrosine (Tyr^416^) in the activation loop replaces the conserved Thr^402^ in Pak2. The differences between threonine and tyrosine phosphorylation in the activation loop will provide a good contrast to study the effects of phosphorylation on the activation loop of Pak2.

In this study, the reciprocal coupling residues from the SCA calculation were mapped onto the Pak1 structure to illustrate their relative positions, and to identify possible interactions and functional effects for these residues. Perturbation of Thr^402^ on Pak2 showed local effects at Val^404^ and Gly^405^ in the activation loop and remote effects through a network centered at Trp^427^. Perturbation of Thr^402^ on the activation loop of Pak2 was similar to that of PKA, while there were significant differences in perturbation with Src Tyr^416(Src)^. Nine reciprocal coupling pairs in Pak2 were identified. The reciprocal coupling residues on the Pak2 surface line up as an activation groove extended from the active site cleft to the large lobe.

## Methods

### Multiple Sequence Alignments (MSA)

The sequences of all 637 human protein kinases were collected from the human kinome database [22]. After eliminating sequences for 106 nonfunctional copies of kinase genes (pseudogenes), 40 atypical kinases, 6 secondary inactive kinase domains, and 3 partial sequences containing less than 200 residues, 482 final sequences were utilized for alignment. We included 47 human kinase domains (pseudo kinases) that lacked some conserved catalytic residues and were predicted to be enzymatically inactive [22], because they have the features of the conserved bilobal structure and increased the diversity of the pool. The collected sequences were aligned using the multiple sequence alignment program, MUSCLE, which processed all sequences at the same time to prevent inconsistencies [30]. The initial alignment from MUSCLE included unnecessary gaps in the target kinase Pak2, expanding the length of sequence about 3-fold. We truncated the gaps to shorten the alignment to 252 residues for the purpose of analysis. The final multiple sequence alignments for the calculation of Pak2 (Figure S1), PKA (Figure S2) and Src (Figure S3) are provided.

### Calculation of Statistical Parameters in MSA

The SCA was calculated as described by Lockless and Ranganathan [1]. The evolutionary conservation parameter (ΔE), based on the 20 amino acid distribution of the human protein kinase alignment, was compared to that of eukaryotic ExPASy database. In MSA, the evolutionary conservation value at each position i was quantitated as a vector of 20 amino acid frequencies and defined as Equation 1.
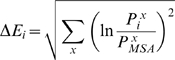
(1)


ΔE values have the arbitrary energy unit, kT*. The unit was omitted to prevent confusion with the real energy unit. X was any of the 20 amino acids. Pi and P_MSA_ were the binomial probability at site i and MSA respectively. The amino acid frequencies in eukaryotic proteins were used as the frequency reference for the probability calculation. The randomness of the amino acid distribution determined the value of evolutionary conservation at each point.

The statistical coupling analysis quantitatively measured the change of the amino acid distribution at site I, caused by conservation of site j in the MSA. The subset, consisting of the sequences with the same amino acid as Pak2 at site j, was collected for coupling analysis. Equation 2 was used to calculate the difference in the amino acid distribution between the subset alignment and MSA (ΔΔE_i,j_)
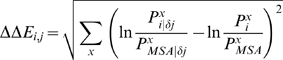
(2)


### Reciprocal Coupling Analysis

Reciprocal coupling analysis was adopted to identify the reciprocal coupling pairs in the results of the SCA, using a 2-dimensional array (282×282). The reciprocal coupling pairs must meet the requirement that when site i was coupled to j, j must couple to i. The top 10 coupling positions for each perturbation site were selected according to their ΔΔE_j,i_ and ΔΔE_i,j_ value. When site i was coupled to j with the highest ΔΔE _j,i_ value, and site j was coupled to i with the highest ΔΔE _i,j_ value, the pairs were identified as the major reciprocal coupling pairs. The statistical coupling data were imported to TIGR Multi-experiment Viewer to generate a matrix of 282×282 [31]. The matrix represented all perturbations in columns to residues in rows. All of the statistical coupling analysis and reciprocal coupling analysis were calculated by Microsoft Excel and Visual Basic for Applications. The structural overlay and graphs were generated by the Swiss-Pdb Viewer [32]. The protein structures used to calculate the distances between residues included 1YHV.PDB (Active Pak1) [21], 1F3M.PDB (Inactive Pak1) [14], 1ATP.PDB (PKA) [24] and 2SRC.PDB (Src) [26].

## Results

### Evolution Value and Conservation of the Residues in Pak2

**Figure 1:**
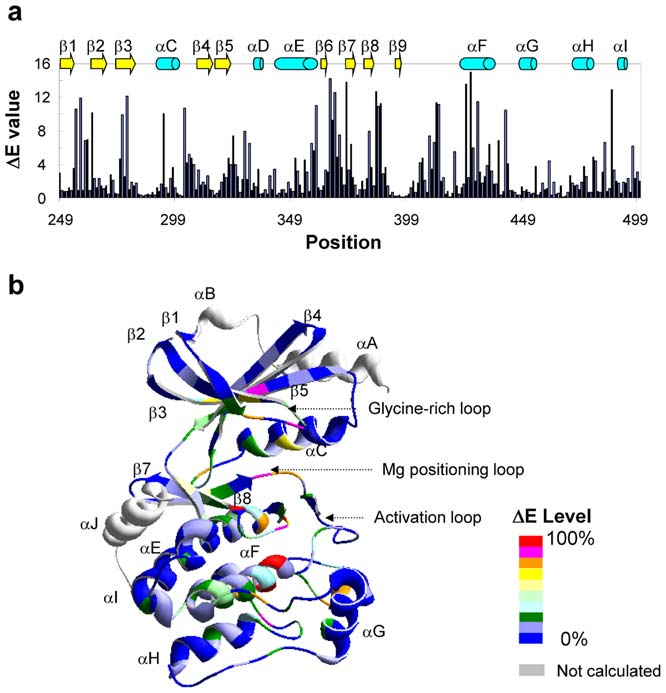
The evolutionary conservation values (ΔE) for Pak2. (a) The Pak2 evolutionary conservation values (ΔE) for amino acids in the catalytic domain (residues 249 to 500). Residue numbers are those for Pak2. (b) The evolutionary conservation values were normalized to percentage and mapped on the tertiary structure of Pak1 (1YHV.PDB), which is highly homologous to Pak2. The levels of ΔE are shown in the color index. The secondary structures, α-helices A-J and β-sheets 1–9, are identified in (b).

Human protein kinases from the kinome were selected for SCA, as indicated in Methods, to calculate the values of the evolutionary conservation (ΔE) for Pak2 (Figure 1a). The ΔE ranged from the least conserved residue Ser^398^ (ΔE = 0.08) to the most conserved Trp^427^ (ΔE = 14.97) in the αF-helix. The percent level of normalized ΔE values were superimposed on the Pak1 structure (Figure 1b). The conserved residues were abundant in the glycine-rich loop, αC-helix, β3-sheet, the activation loop, the magnesium positioning loop and αF-helix. This indicates the universal importance of the residues related to catalytic activity, ATP binding and magnesium ion binding. The αF-helix shown as a highly conserved region may play an important role in the catalytic activity and maintaining the structure of the large lobe.

**Figure 2:**
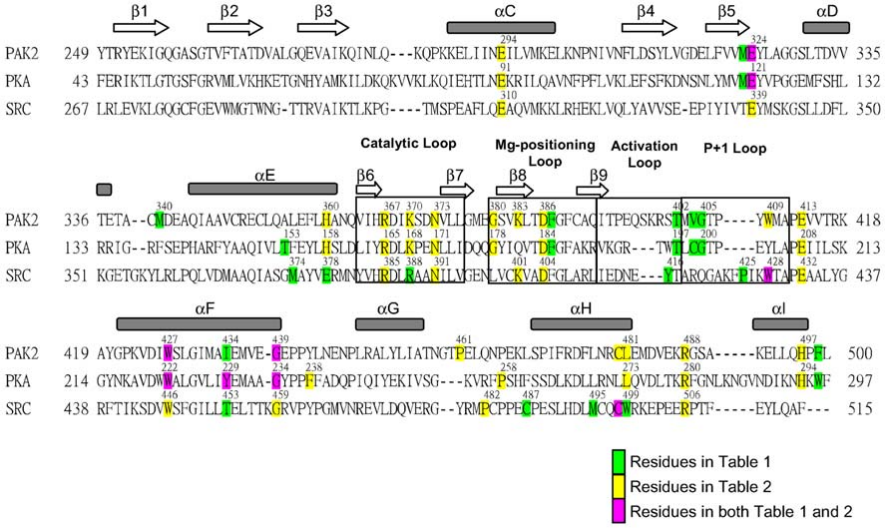
Alignment, secondary structure, and functional elements of the catalytic subunits of Pak2, PKA and Src. Secondary structure assignments are shown as rectangles (α-helices) and arrows (β-strands) above the aligned sequences. The assigned functional domains are shown by the empty box in the alignment. The residues highlighted in green are the ones appeared in Table 1; the residues highlighted in yellow are the ones appeared in Table 2; the residues highlighted in pink are the ones appeared in both Table 1 and 2.

The detailed sequence alignment, secondary structures and domains of Pak2, PKA and Src are shown in Figure 2. The glycine-rich loop, catalytic loop, magnesium positioning loop, APE motif and F-helix are highly conserved in the alignment. The secondary structures and the highly conserved regions became important checkpoints for the multiple sequence alignment. The residues perturbed by the phosphorylation site in the activation loop and the reciprocal coupling residues of Pak2, PKA and Src are also identified in the alignment.

### Evolutionary Coupling Network of Thr^402^ for Pak2

**Figure 3:**
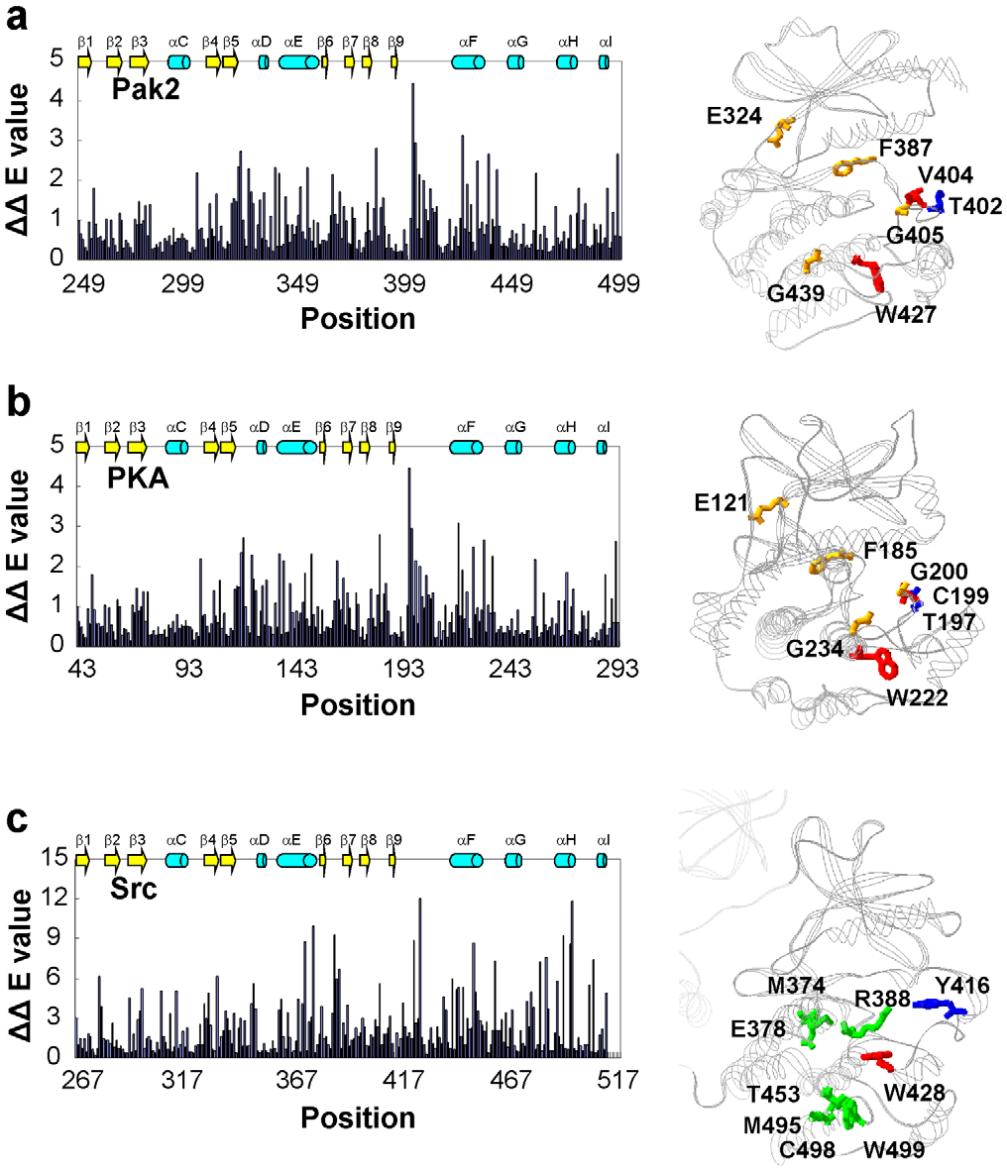
The residues of Pak2, PKA and Src statistically coupled to the conserved phosphorylation site in the activation loop. (a) The histogram represents the statistical coupling energy (ΔΔE) of the Pak2 catalytic domain (residues 249 to 500) perturbed by Thr^402^ in the activation loop. The stick representation of the perturbation effect of Thr^402^ (blue) displays Pak2 residues with ΔΔE>3.0 (red) and between 3.0 and 2.6 (orange). (b) The histogram represents the statistical coupling energy (ΔΔE) of the catalytic subunit domain of PKA (residues 43 to 297) perturbed by Thr^197^ in the activation loop. The results are superimposed on the x-ray crystal structure of PKA (1ATP.PDB). The color chart is the same as Figure 2. The phosphorylation site Thr^197^ (blue), the major coupled residues Cys^199^ and Trp^222^ (ΔΔE>3.0, red), and the secondary coupled residues (ΔΔE between 3.0 and 2.6, orange) are identified. (c) The histogram represents the statistical coupling energy (ΔΔE) of the Src catalytic domain (residues 267 to 515) perturbed by Tyr^416^ in the activation loop. The statistical coupling residues of Src are shown on the tertiary structure (2SRC.PDB). The phosphorylation site Tyr^416^ (blue), the center of the coupled residues Trp^428^ (ΔΔE = 12.06, red), and the secondary coupled residues (ΔΔE between 12.0 and 7.6, green) are identified.

The evolutionary coupling values (ΔΔE) of the residues to the autophosphorylation site Pak2 Thr^402^ was quantified by perturbation to the conservation value ΔE [1]. We calculated the changes of the evolutionary coupling values (ΔΔE) of each individual Pak2 residue caused by perturbation at moderately conserved Thr^402^ (34.6%). The results of perturbation at Thr^402^ (Figure 3a, left panel and Table 1) were positioned on the crystal structure of the homologous Pak1 to visualize the perturbation effect in terms of the color index (Figure 3a, right panel). The average of ΔΔE for all residues was 0.83. Local effects caused by Thr^402^ perturbation identified Val^404^ (ΔΔE_404V,402T_ = 4.43) and Gly^405^ (ΔΔE_405G,402T_ = 2.95) in the peptide positioning loop. The second strongest effect was observed distally at Trp^427^ (ΔΔE_427W,402T_ = 3.12) in the F-helix (residues 423–438). Additionally, Phe^387^ (ΔΔE_387F,402T_ = 2.80) in the magnesium positioning loop was affected by perturbation of Thr^402^. This suggests a communication pathway between Thr^402^ in the activation loop, the magnesium positioning loop and the F-helix.

**Table 1:** Top ten residues and statistical coupling energy for the perturbation of Thr^402^ for Pak2, Thr^197^ perturbation for PKA, and Tyr^416^ for Src.

Pak2		PKA		Src	
Residue	ΔΔE_i,402T_	Residue	ΔΔE_i,197T_	Residue	ΔΔE_i,416Y_
Val404	4.43	Cys199	4.45	Trp428	12.06
Trp427	3.12	Trp222	3.10	Trp499	11.80
Gly405	2.95	Gly200	2.95	Glu378	9.94
Phe387	2.80	Phe185	2.80	Arg388	9.28
Glu324	2.72	Glu121	2.71	Met495	9.21
Gly439	2.66	Gly234	2.65	Pro425	8.85
Phe499	2.65	Trp296	2.63	Met374	8.77
Ile434	2.48	Tyr229	2.50	Thr453	8.69
Met323	2.34	Met120	2.34	Cys498	8.58
Met340	2.33	Thr153	2.31	Cys487	7.61

To compare the perturbation of threonine in the activation loop of Pak2, two other protein kinases, cAMP-dependent protein kinase (PKA) and Src (in the AGC and TK protein kinase hierarchical groups, respectively), were also examined by SCA. Phosphorylation of Thr^197(PKA)^ in PKA and Tyr^416(Src)^ in Src in the activation loop is essential for their protein kinase activity [15], [26], [27], [33], [34]. SCA was applied to perturbation of Thr^197(PKA)^ in PKA (34.8%) and Tyr^416(Src)^ in Src (20.5%). The average of ΔΔE_i,197T_ for all residues of PKA was 0.83, and the average of ΔΔE_i,416Y_ for Src was 2.31. The results with Thr^197(PKA)^ in PKA showed a similar pattern of affected residues as Pak2, as visualized in the structure of PKA (1ATP.PDB) (Figure 3b). Cys^199(PKA)^ (ΔΔE_199C,197T_ = 4.45) of PKA was two residues away from the phosphorylation site Thr^197^, which is the same as Val^404^ and Thr^402^ in Pak2. Tyr^416 (Src)^ in the activation loop of Src resulted in similar coupling residues in Pak2. These residues were superimposed to the structure of Src (2SRC.PDB) (Figure 3c). Trp^428(Src)^ (Trp^409^ in Pak2) in the peptide positioning loop was the most impacted residue by Tyr^416(Src)^ perturbation (ΔΔE_428W,416Y_ = 12.06), different from Pak2 and PKA.

### Reciprocal Coupling Analysis of Pak2

**Figure 4:**
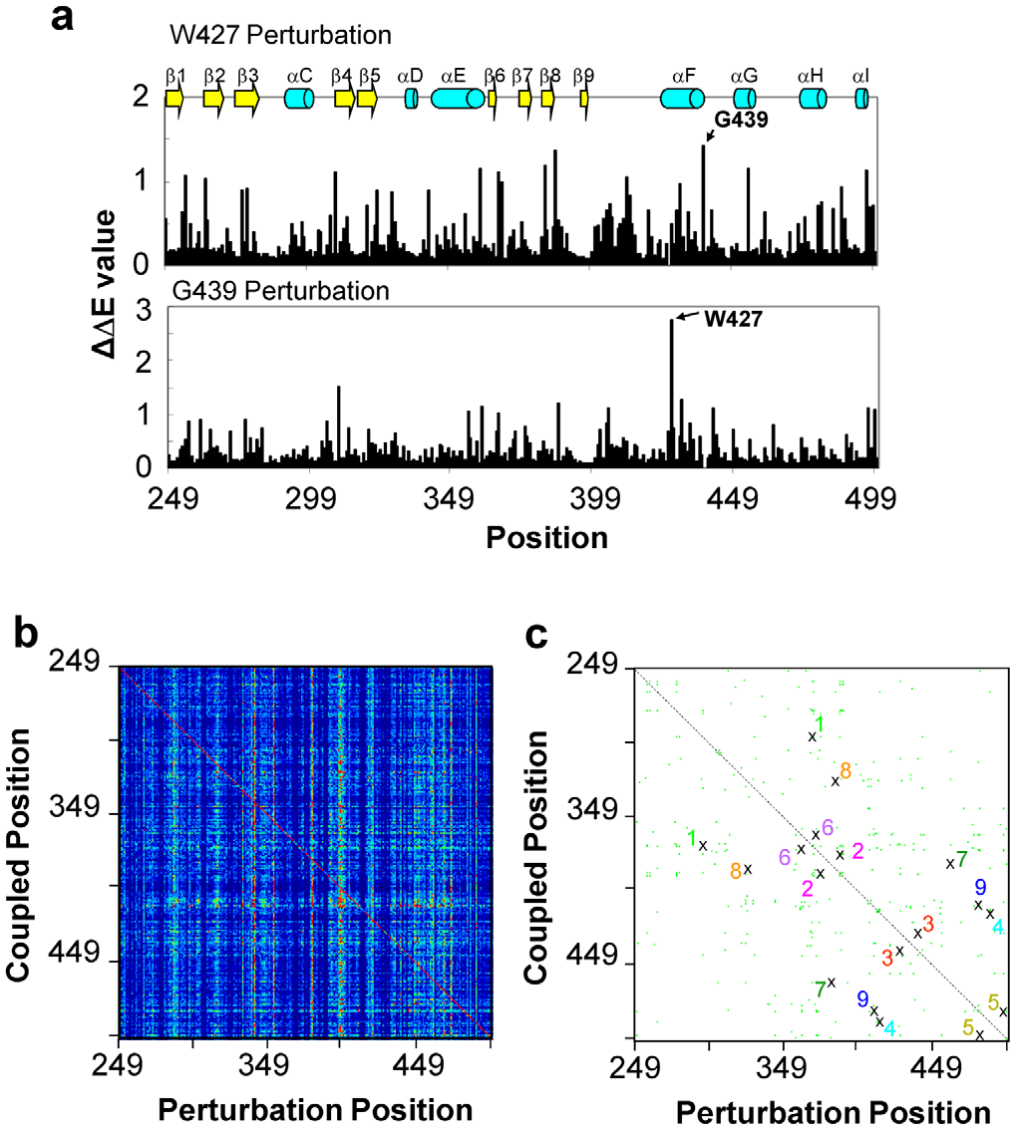
Evolutionary reciprocal coupling of Pak2. (a) Perturbations of Trp^427^ (upper panel) and Gly^439^ (bottom panel) were generated to obtain statistical coupling energies to all of the Pak2 residues. The most impacted residue of Trp^427^ perturbation is Gly^439^ and visa versa, and is defined as the major reciprocal coupling pair. (b) The results of the coupling energy (ΔΔE) calculations were imported into TIGR MeV program to show the 252 (Perturbation)×252 (Coupling) matrix. The intensity of ΔΔE was expressed in the heat map color scheme shown on top of the graph. (c) Plot of reciprocal coupling residues. The cross marks show the major reciprocal coupling residues (the highest ΔΔE_i,j_ and ΔΔE_j,i_ values in each perturbation) and the green dots show the minor reciprocal coupling residues (top 2–10 ΔΔE_i,j_ and ΔΔE_j,i_ values in each perturbation). The nine reciprocal coupling pairs are identified in Table 1. The dashed line is a symmetrical line for the coupling pairs and measures the length between residues.

To analyze the evolutionary coupling effect on one residue, we mutated the particular residue *in silico* and measured the evolutionary coupling values (ΔΔE) of each individual Pak2 residue. Two examples of mutations at Trp^427^ and Gly^439^ are shown in Figure 4a. The ΔΔE values indicate the extent of perturbation caused by the mutation. In these two examples, Gly^439^ (ΔΔE_439G,427W_ = 1.5) was the strongest coupling residue to Trp^427^ (Figure 4a, upper panel); Trp^427^ was the strongest coupling residue to Gly^439^ in (Figure 4a, lower panel). Thus, Trp^427^ and Gly^439^ are reciprocally coupled through evolution. The complete calculations for each position are shown in the two-dimensional matrix (Figure 4b).

Reciprocal coupling analysis of the entire catalytic domain of Pak2 was carried out to retrieve the reciprocal coupling pairs. The top 10 evolutionary coupling values (ΔΔE) were extracted from Figure 4b for reciprocal coupling analysis and the data are shown as green dots (Figure 4c). A total of nine reciprocal coupling pairs with the strongest correlation between two residues were identified, as indicated by pair number (Figure 4c, Table 2). The generated map of the reciprocal coupling pairs was symmetrical along the diagonal.

**Table 2:** The common and specific coupling pairs from reciprocal coupling analysis.*

Common Coupling	Pak2 Residues	Distance	PKA Residues	Distance	Src Residues	Distance	Pair #
	E294-R367	10.1 Å	E91-R165	10.2 Å	E310-R385	9.6 Å	1
	N373-D386	3.6 Å	N171-D184	3.1 Å	N391-D404	2.9 Å	2
	W427-G439	14.8 Å	W222-G234	14.8 Å	W446-G459	15.0 Å	3
	E413-R488	2.6 Å	E208-R280	2.8 Å	E432-R506	2.7 Å	4
**Specific Coupling**	L481-H497	10.1 Å	L273-H294	10.1 Å			5
	H360-K370	11.8 Å	H158-K168	12.4 Å			6
	G380-P461	25. 3Å	G178-P258	22.5 Å			7
	E324-K383	3.2 Å			E339-K401	2.7 Å	8
	W409-C480	8.8 Å			W428-C498	8.6 Å	9
			Y229-F238	7.4 Å			10

* The common coupling pairs exist in Pak2, PKA and Src. The specific coupling pairs exist in either one or two protein kinases.

The distance between the two coupling residues is defined as the closest distance in the X-ray crystal structures 1YHV.PDB, 1ATP.PDB and 2SCR.PDB, as measured using the Swiss-Pdb Viewer.

**Figure 5:**
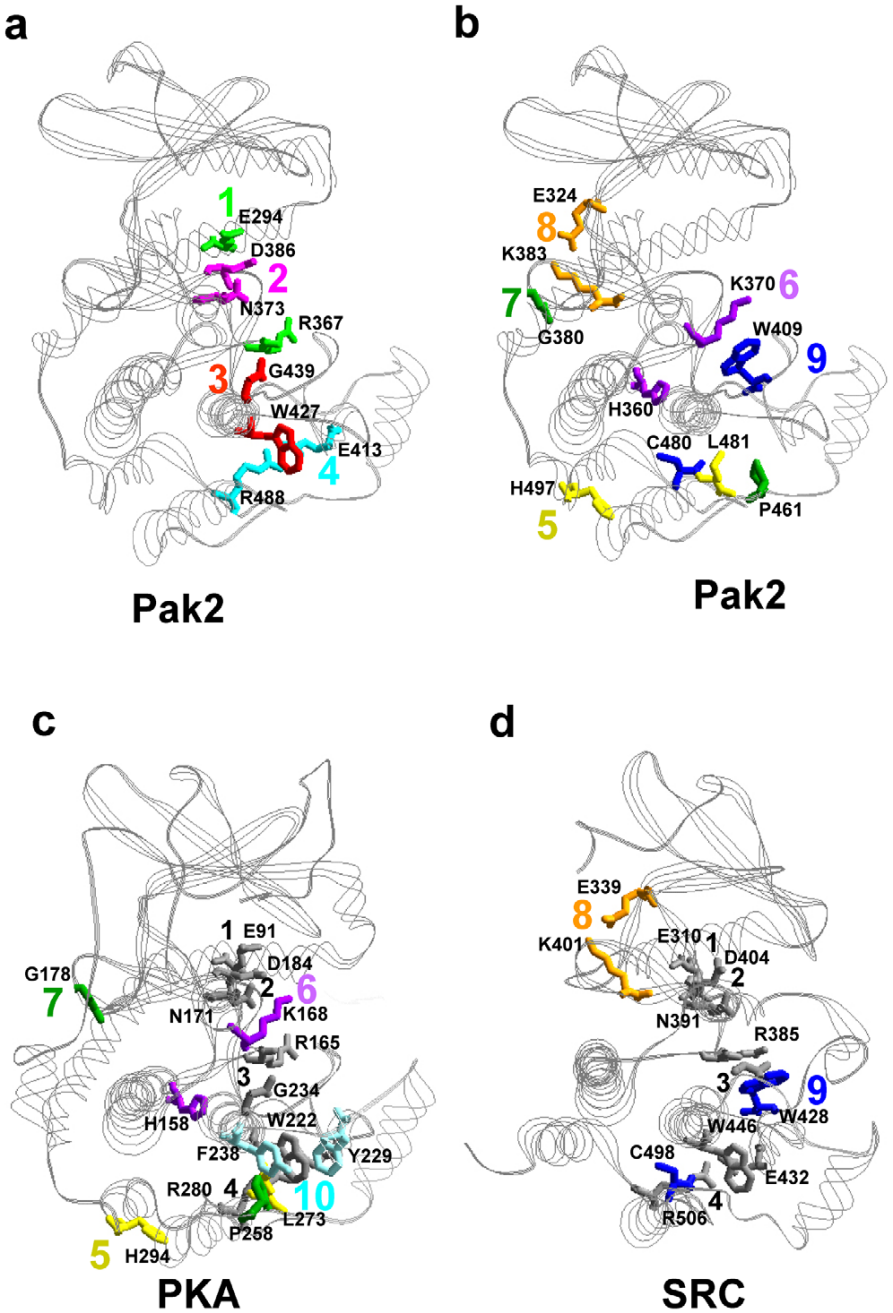
Visualization of the reciprocal coupling pairs for Pak2, PKA and Src. (a) Four common reciprocal coupling pairs for Pak2 were superimposed on the Pak1 structure (1YHV.PDB) (see Table 1). (b) Five specific reciprocal coupling pairs were superimposed on the same structure. (c) Four specific reciprocal coupling pairs for PKA were identified on the PKA structure (1ATP.PDB). (d) Two specific reciprocal coupling pairs in Src were shown on 2SRC.PDB. The same color was used when two residues were reciprocally coupled. In (c) and (d), the common reciprocal couplings between the three structures are in gray.

The nine reciprocal coupling pairs of Pak2 were positioned on the structure for Pak1 (1YHV.PDB) (Figure 5a,b). Among the nine pairs, three had residues that interacted with each other or were within the contact distance. Asn^373^- Asp^386^ (Pair 2), Glu^413^- Arg^488^ (Pair 4) and Glu^324^- Lys^383^ (Pair 8) were within 3.6 Å (Table 2). Pair 4 and Pair 8 were identified as ion pairs. From the crystal structures (Figure 5a,b), Glu^413^- Arg^488^ (Pair 4) stabilized the distance between the peptide positioning loop and the H-helix in the large lobe. Pair 8 is an ion pair in the hinge region of the bilobal structure. Pair 2 (residues Asn^373^ and Asp^386^) were located in the active site cleft.

On the other hand, the rest of the residues were not within a contactable distance, but were evolutionarily conserved. The correlation between these residues may be caused by structural or functional structures. Glu^294^- Arg^367^ (Pair 1) was solvent exposed in the active site cleft and both residues were important for stabilization of phosphate on ATP and the activation loop [21], [24], [25], [33], [34]. The hydrophobic F-helix, which stabilizes the large lobe, is part of the hydrophobic pocket critical for PKA activity [35]. Evolutionary coupling of Trp^427^ and Gly^439^ (Pair 3) in the F-helix of Pak2 (same in PKA) could also provide stabilization of the large lobe. However, the function of four of the reciprocal coupling pairs was not clear in the initial analysis. His^360^- Lys^370^ (Pair 6), 11.8 Å apart and located between the D-helix and the catalytic loop, could be stabilizing the catalytic loop region. Trp^409^-Cys^480^ (Pair 9) in the peptide positioning loop and H-helix, and Leu^481^- His^497^ (Pair 5) between the H- and I-helices, were 8.8 and 10.1 Å, respectively. Together, they could stabilize the region containing the peptide positioning loop, the H-helix and the I-helix. The two residues, Gly^380^- Pro^461^ (Pair 7) were at a remote distance (25.3 Å), and associated with two regions, the C-terminus and the G-H loop.

### Comparison of Reciprocal Coupling Pairs between Pak2, PKA and Src

The reciprocal coupling pairs of Pak2, PKA and Src were compared (Table 2) to seek the required and universal coupling pairs in protein kinases. The reciprocal pairs were separated in two groups, common coupling pairs and specific coupling pairs. There were four common reciprocal coupling pairs for all three of the protein kinases. The specific coupling pairs appeared only in one or two proteins. Pak2 had five specific reciprocal coupling pairs, PKA had four and Src had two. Three specific coupling pairs from PKA and two from Src were present on Pak2.

The distances between the specific evolutionary coupling pairs in Pak2 were from 2.6 Å to 14.8 Å and with the average of 7.7 Å. There were minor differences in the average distances of the common coupling pairs among Pak2 (7.7 Å), PKA (7.7 Å) and Src (7.5 Å). Because these four common coupling pairs are conserved in Pak2, PKA and Src, it they could be responsible for the general and critical functions of protein kinases, such as the catalytic events in ATP binding, magnesium binding or protein substrate binding. Interestingly, these residues are localized in a two-dimensional surface extending from the active site to the F-helix (Figure 5a). The core structure of the large lobe is composed of six helices and several loops between the helices (Figure 1b). The D, E, G, H and I helices were surrounding the hydrophobic F-helix. The catalytic sites, the magnesium positioning loop and the activation loop resided on top of the core. Most coupling residues were on the interface between the helix-core (D, E, F, H, I helices) and the activity region, including the magnesium positioning loop, the activation loop, and the peptide positioning loop. The G-helix on the other side is in charge of the enzymatic activity (Figure 1b). The residues of the G-helix have low ΔE values, although the helix is conserved in the known kinase structures. The activation loop and G-helix are important regulatory domains [23], [33], [34]. The peptide positioning loop is anchored by the coupling of Glu^413^-Arg^488^ to the H-helix.

The specific coupling pairs for Pak2, PKA and Src are shown in the crystal structures (1YHV.PDB, 1ATP.PDB and 2SRC.PDB respectively) (Figure 5b, c, d). The specific reciprocal coupling pairs included both distant pairs and proximal pairs. The distances between the specific evolutionary coupling pairs in Pak2 were from 3.2 Å to 25.3 Å and with the average of 11.8 Å. The average distances of the specific coupling pairs are 13.1 Å for PKA and 5.6 Å for Src (Table 2).

The Pak2 catalytic domain contained all of the evolutionary coupling pairs of PKA and Src, except for Tyr^229^- Phe^238(PKA)^ in PKA (Pair 10), which was unique. Tyr^229^- Phe^238(PKA)^ (Pair 10), existing only in PKA, appeared in two approximately parallel (9°) ring-structures in the F-helix and the F-G loop. These two residues were 7.35 Å apart (7.48 Å when PKI was bound) [24], [36]. Interestingly, when the regulatory subunit bound to the G-helix of the catalytic subunit, the two ring structures were twisted (16°) and moved closer to each other (7.17 Å) [37]. Thus, an interaction between Tyr^229(PKA)^ and Phe^238(PKA)^ might be involved in regulation of G-helix of PKA, which would be different from that of Pak2 and Src. Pairs 5, 6 and 7 in Pak also existed in PKA, but not in Src. With Src, Glu^339^- Lys^401(Src)^ (Pair 8) and Trp^428^- Cys^498(Src)^ (Pair 9), were the two specific coupling pairs and were present in Pak2, but not PKA. Glu^324^- Lys^383^ (Pair 8) was ion paired in the hinge region of Pak2 and Src with the side chains contacting each other. Activation of Pak does not destroy the interaction between Glu^324^ and Lys^383^, but Lys^383^ is twisted in the active conformation. Trp^428(Src)^ in Pair 9 was significantly perturbed by Tyr^416(Src)^, indicating this reciprocal coupling pair was related to tyrosine phosphorylation at the activation loop. When we examine further these specific coupling pairs, at least one residue of the coupling pairs 5, 6, 7, and 9 are in contact with the F-helix, indicating the importance of this F-helix.

## Discussion

The protein kinase Pak2 can be activated by either Cdc42 binding or caspase 3 cleavage. Cdc42 binds to the Cdc42/Rac interaction and binding sequence (CRIB) (residues 74–87) of Pak2, which overlaps with the autoinhibitory domain (AID). Binding of Cdc42 to inactive Pak2 disrupts the inhibition [7]. Under apoptotic stress, Pak2 is constitutively activated by caspase 3 cleavage and autophosphorylation [8]. In either event, the protein kinase activity of Pak2 requires ATP binding, autophosphorylation of Thr^402^ and disruption of the autoinhibition to obtain an active conformation. In our previous study, we used amide hydrogen/deuterium exchange and mass spectrometry to analyze the inactive and active Pak2. The N-terminus, glycine-rich loop, C-helix, magnesium positioning loop, F-helix and the G-helix are affected by the activation of Pak2. In order to analyze further the activation mechanism of Pak2, we have now used statistical methods to systematically analyze the critical residues in the protein kinases through evolution.

**Figure 6:**
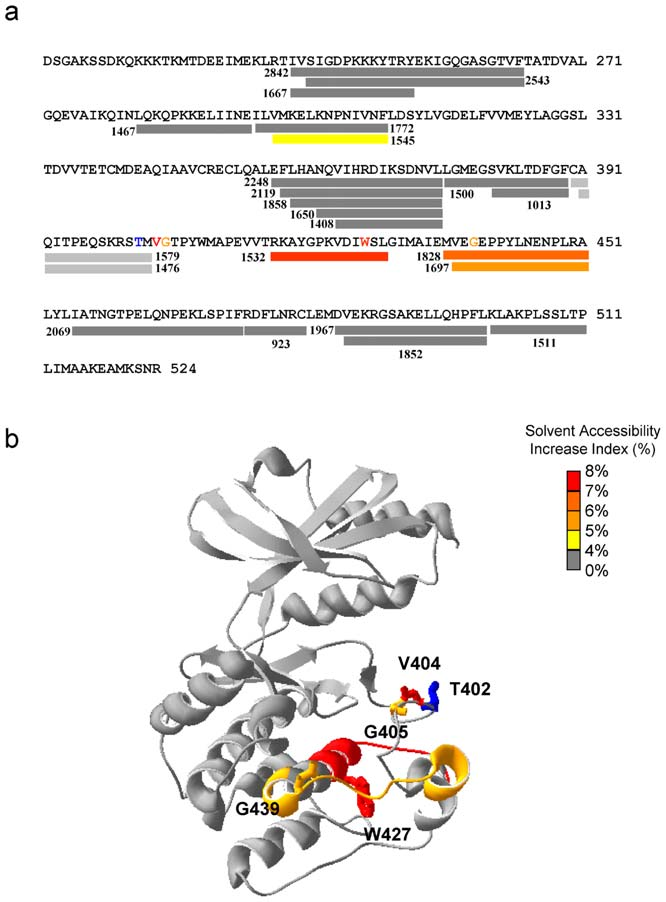
Differences in solvent accessibility between caspase-cleaved non-phosphorylated and autophosphorylated Pak2. (a) The colored bars illustrate the solvent accessibility changes in the primary sequence. The numbers show the mass (M/Z) of the peptic peptides with a measurable MALDI-TOF signal after H/D exchange. An increase of solvent accessibility following autophosphorylation is shown as the solvent accessibility index. Two fragments (light gray bars), m/z 1476 and 1579, containing Thr^402^ disappeared after phosphorylation. (b) The differences in solvent accessibility are shown on crystal structure of Pak1 (1YHV.PDB). Residues, Trp^427^ (red) and Gly^439^ (orange), were statistically coupled to Thr^402^ (blue). Trp^427^ and Gly^439^ were in the fragments m/z 1532 and 1828 that had increased solvent accessibility after autophosphorylation.

Phosphorylation of Thr^402^ in the activation loop has been regarded as a crucial element for conformational change in the activation loop and is required for Pak2 activity. To examine the Thr^402^ perturbation effect, we compared the evolutionary coupling analysis with the amide H/D exchange data obtained during activation of Pak2 [23] (Figure 6). Autophosphorylation of Pak2 caused a significant increase in the deuterium exchange in the region 417–429 containing part of the F-helix and the E-F loop, and the region 436–451 containing parts of the F and G-helices and the F-G loop. In the SCA data, we have shown Val^404^ and Gly^405^ coupled locally with Thr^402^, and remotely with Trp^427^ and Gly^439^ in the F-helix in response to autophosphorylation of Thr^402^. Also, Trp^427^ and Gly^439^ are reciprocal coupling pairs. Perturbation of the critical autophosphorylation site Thr^402^ showed the importance of the conserved Trp^427^ in the F-helix.

Using statistical analysis, Kannan and Neuwald [38] proposed the F-helix as the most conserved region of protein kinase family. The tryptophan in the F-helix played a key role in proper positioning of the G-helix, coordinating with a buried water molecule and forming a hydrophobic pocket below the F-helix [38]. Kornev et al [39] showed this hydrophobic pocket involves hydrophobic residues in the F-helix and four conserved leucines in the H-helix. Kannan et al [40] have shown further that the eukaryotic protein kinases have the regulatory machinery that is defined by the G-Helix and the activation loop, and is missing in the prokaryotic eukaryotic-like kinases. Phosphorylation of the activation loop couples this regulatory machinery to the core catalytic machinery in the C-terminal lobe. In our reciprocal coupling analysis, the F-helix serves as a central helix in contact with most of the reciprocally coupled residues. Particularly, Leu^481^, Cys^480^, Arg^488^, Pro^461^, Trp^409^ are within 5 Å of Trp^427^ (Fig 5a,b). Among these residues, Trp^427^ in the F-helix, and Leu^481^ and Cys^480^ in the H-helix were the main components of the hydrophobic pocket [39]. The hydrophobic F helix has been identified as a mechanism for assembling an active protein kinase and for anchoring the two hydrophobic spines that link both the N-lobe and the C-lobe [39], and is an independent validation of the importance of the F-Helix. It also explains in part how some of these distal residues may be functionally correlated. For instance, many of the correlated residues such as Phe^185(PKA)^ are part of the spine, and many of the correlated residues (Fig 5c) are directly adjacent to the regulatory spine.

Threonine phosphorylation on the activation loop of Pak2 perturbed the kinase using a similar network as PKA, but a different network than the tyrosine phosphorylation on Src. Perturbation at the threonine autophosphorylation site on the activation loop of Pak2 identified evolutionary coupled Val^404^ and Gly^405^ in the peptide-positioning (P+1) loop, Trp^427^ and Gly^439^ in the F-helix and Met^323^ and Glu^324^ in the hinge region. On the other hand, perturbation of the Src Tyr^416(Src)^ phosphorylation site was coupled with Pro^425(Src)^ and Trp^428(Src)^ in the peptide positioning loop and Met^495(Src)^, Cys^498(Src)^ and Trp^499(Src)^ in the H-helix. The peptide positioning loop binds to the protein substrates for phosphorylation and its sequence is important for the recognition of specific substrates [33], [34]. With the different residues in this region (Table 1 and Figure 2), the phosphorylation of the tyrosine in the activation loop of Src could perturb different residues in the peptide positioning loop as compared to that of the threonine in Pak2 and PKA. The differences relate directly to the mechanism for recognition of the P-site residue in the protein substrate. In the case of the two serine/threonine kinases, Gly^200(PKA)^ or Gly^405(Pak2)^ is binding directly to the backbone of the P-site residue that is to be phosphorylated. In the case of the tyrosine kinases the backbone is too far away and the peptide is positioned instead by the aromatic ring and stacking with the aromatic ring in the P+1 loop. Thus, the two Ser/Thr kinases, Pak2 and PKA, have a different substrate recognition mechanism than the tyrosine kinase Src. Moreover, the hinge region was shown to be significantly altered between the open and closed conformation of the kinase domain of Pak2 and PKA [14], [21], [24], [25]. Phosphorylation of Pak2 and PKA may affect the hinge region, leading to the movement between the two lobes of the kinase domain. Pak2 and PKA alter activation via the residues next to the phosphorylation site in the activation loop, the hinge region and F-helix while Src is via Pro^425(Src)^ and Trp^428(Src)^ on the peptide positioning loop, the catalytic loop and H-helix (Table 1, Figure 3). Thus, there are two different ways to achieve the kinase catalytic activity.

Recently, several studies have used statistical analysis and structural comparison to identify critical residues in protein kinases [38]–[41]. The critical roles of the F-Helix, Trp^222(PKA)^ (Trp^427^ in Pak2) in the F-Helix and the hydrophobic pocket between the F and H-helix for the protein kinases were particularly identified in these studies. Two clusters of residues were proposed to be evolutionary coupled [41]. Perturbation of the phosphorylation site Thr^197(PKA)^ (Thr^402^ in Pak2) affected several proposed coupling residues. Trp^222(PKA)^ in F-helix of PKA (Trp427 in Pak2) is claimed as a center in one of the two clusters of evolutionary coupled residues, while Cys^199(PKA)^, Gly^200(PKA)^, Gly^234(PKA)^, Trp^296(PKA)^ and Tyr^229(PKA)^ in PKA were in the other cluster of residues. These reports support our finding that Trp^222^ in PKA and Trp^427^ in Pak2 is a key interaction site with Thr^197(PKA)^ in PKA and Thr^402^ in Pak2. We show that phosphorylation of this critical threonine regulates critical structural changes that lead to activation of the protein kinases.

**Figure 7:**
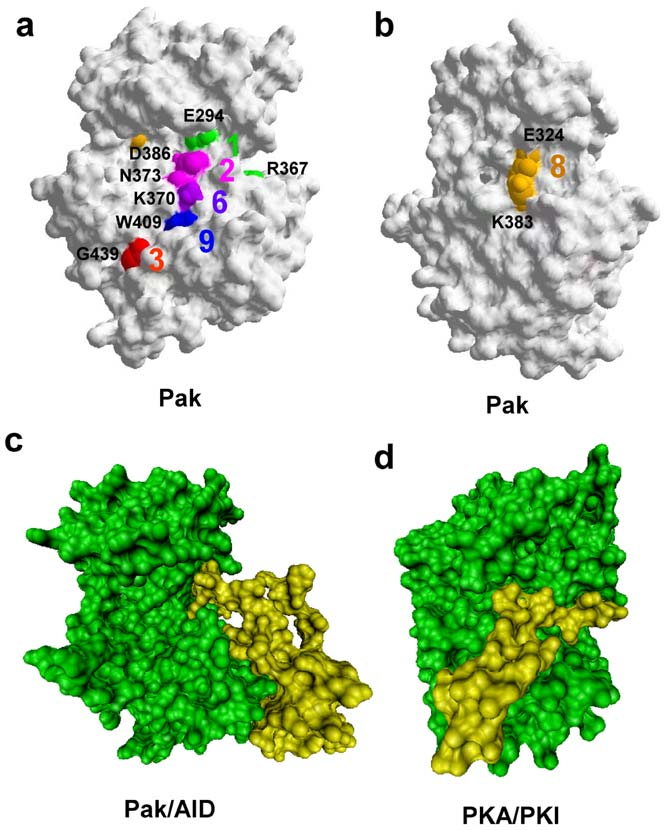
The protein surface representation of Pak2 and PKA, identification of reciprocal coupling pairs, and association with inhibitory peptides. (a) Identification of the Pak2 reciprocal coupling residues that are on the Pak1 surface (1YHV.PDB). (b) The structure turned 120 degrees counterclockwise from (a) shows the reciprocal coupled ion pair in the hinge region. (c) The catalytic domain of Pak (green) binding to the AID (yellow) modified from 1F3M.PDB. (d) PKA (green) bound to PKI (yellow) modified from 1ATP.PDB.

The residues exposed to the solvent are most likely to contact with ligand, substrate or other proteins. All of the reciprocal coupling residues having an exposed side chain on the protein surface of the Pak structure are identified in Figure 7a, b. The coupling residues on the surface of Pak2 lined up in a groove extending from the active site cleft to the large lobe. The residues residing in the groove include Glu^294^ and Arg^367^ (Pair 1) and Asn^373^ and Asp^386^ (Pair 2) in the active site cleft. Lys^370^ in Pair 6, Trp^409^ in Pair 9 and Gly^439^ in Pair 3 are on the large lobe (Figure 7a). Glu^324^ and Lys^383^ (Pair 8) are exposed on the Pak2 surface in the hinge region (Figure 7b). Lys^370^, Trp^409^ and Gly^439^ in the large lobe, have their reciprocal coupling partners, His^360^, Cys^480^ and Trp^427^ below the surface of Pak.

Glu^294^- Arg^367^ (Pair 1) and Asn^373^- Asp^386^ (Pair 2) are the two reciprocal coupling pairs in the active site. These four conserved residues are responsible for the stabilization of the magnesium ions and ATP, the C-helix and the activation loop in PKA [33], [34]. The side chains of Glu^294^ and Arg^367^ in the inactive conformation of Pak1 (1F3M.PDB) interact with the kinase inhibitory region (residues 136–146 for Pak2, 138–147 for Pak1) of the autoinhibitory domain [14]. While in the active conformation (1YHV.PDB), the relative positions of Glu^294^ and Arg^367^ are significantly changed due to the twist in the C-helix (Fig 5a) [21].

In the inactive Pak1 conformation (1F3M.PDB), Asn^373^ and Asp^386^ (Pair 2) are solvent exposed. These two residues are coordinated with manganese ions in the PKA structure (1ATP.PDB). In our previous H/D exchange experiments, we found that AID binding to Pak2 does not block coordination of the magnesium ions to Pair 2 [23]. A comparison of Figure 7c to Figure 7a indicates the AID blocks Pair 1, while Pairs 2, 3, 6 and 9 are still solvent exposed. When we overlaid the Pak1 and PKA structures, the groove overlapped with the pseudosubstrate (PKI) binding region on PKA (Figure 7d) [24]. When the contact region between the PKA catalytic domain and the regulatory domain was compared with the Pak2 coupling residues, part of the contact region resided in the PKI binding groove, thus blocking substrate binding. We predict that variation of the amino acid composition of the protein kinases in this groove will alter the substrate specificity.

Note that several residues clustered within the hydrophobic core contacting to the F-helix beneath the surface were also shown to be involved in the substrate binding affinity of PKA [42], [43], including Trp^222(PKA)^ (Pair 3), Glu^208(PKA)^ and Arg^280(PKA)^ (Pair 4) and His^294(PKA)^ (Pair 5). These identified residues stretched from the active site of the enzyme to the C-terminal substrate-binding domain. Coupling of the active site to the H and I helices was independently correlated and then experimentally validated in a genetic screen by two-hybrid assays [42], [43]. Arg^280(PKA)^ between the H and I-helix formed a salt bridge interaction with Glu^208(PKA)^ in the APE motif packing up against Trp^222(PKA)^. Mutation of Arg^280(PKA)^ to lysine decreased the kinase activity in PKA [44].

On the reverse side of Pak2, Glu^324^- Lys^383^ (Pair 8) are ion paired in the hinge region of Pak2 and of Src, with their side chains contacting each other (Figure 7b). This solvent exposed pair connects the linker region of the bi-lobal structure with the magnesium positioning loop [14], [21], [26]. The two lobes of the active conformation (1YHV.PDB) are closer than the inactive conformation (1F3M.PDB) of Pak1. The active conformation has a 15 degree rotation of the bilobal structure, compared to the inactive conformation [21]. When we examined Pair 8 in both structures, the hydrogen bonds between Glu^324^ and Lys^383^ were twisted in the inactive conformation. In this inactive conformation, the distances between two oxygen atoms of Glu^324^ and the nitrogen atom of Lys^383^ are 3.96 and 2.87 Å, while the ion pair shows shorter distances, 3.25 and 2.89 Å in the active conformation.

## Supporting Information

Figure S1 Multiple sequence alignment of human protein kinases based on Pak2.
(0.13 MB TXT)

Figure S2 Multiple sequence alignment of human protein kinases based on PKA.
(0.13 MB TXT)

Figure S3 Multiple sequence alignment of human protein kinases based on Src.
(0.13 MB TXT)
